# Editorial on the Special Issue: “Pancreatic Islets of Langerhans: Not Only Beta-Cells”

**DOI:** 10.3390/biom11111646

**Published:** 2021-11-07

**Authors:** Roberta Malaguarnera, Salvatore Piro

**Affiliations:** 1 Faculty of Medicine and Surgery, “Kore” University of Enna, 94100 Enna, Italy; roberta.malaguarnera@unikore.it; 2 Department of Clinical and Experimental Medicine, Internal Medicine, Garibaldi-Nesima Hospital, University of Catania, 95122 Catania, Italy

This year marks the centenary of the discovery of insulin. The year 1921 was the beginning of a new era for the world of diabetology, promising life to all insulin-deficient patients. The discovery of insulin has been the most important event that has changed the world of scientific research [1]. For this reason, it deserves to be remembered. In 1923, just two years after the discovery of insulin, the existence of glucagon was first reported [2].

In those years, however, the Nobel Prize for the discovery of insulin had already been awarded, and this second pancreatic hormone has always remained *“the second”*, “the son *of the servant”*, *“the follower”*, *“the wingman”*, or *“the antidote for hypoglycemia”*. Despite this, the history of glucagon, although less cutting edge than that of insulin, represents a milestone in medicine. From the purification of glucagon (1955) to its first commercialization (1957), “the son of the servant” has always held a discrete appeal. In the 1970s, underground literature focusing on the “second pancreatic hormone”, appeared in the scientific world [3,4,5]. The studies by Roger Unger raised the doubt that “the son of the servant” had a much greater role rather than a simple antidote for hypoglycemic crises. With this pioneering and revolutionary literature, there arose a new concept according to which gut and pancreas cross-talked and the peptides derived from proglucagon could play an important role in the pathophysiology of type 2 diabetes mellitus (T2DM). This different point of view could have rewritten the history of this metabolic disease. However, it was the 1970s: the Beatles were already well known, John Lennon composed “Imagine” and man had already been to the moon. Nobody was willing to change their own idea; no one was ready to change their own perspective and point of view again. Insulin was sufficient to be a pillar of the history of diabetes; it was enough for us to consider the pancreatic islet as synonymous for beta cells, and therefore, for insulin. At that time, there was no need to understand why this perfect little organ showed other cells, albeit to a lesser extent than insulin-producing beta cells. In 1986, however, the description of the incretin effect in humans managed to undermine these consolidated and unshakable certainties that had been held until then [6]. The physiological phenomenon that triggers a greater secretion of insulin following the ingestion of food, finally also took into greater consideration the effect of glucagon secretion and its role in glucose homeostasis and in T2DM pathophysiology. Although the incretin effect was described as belonging to GLP-1 (glucagon Like Peptide 1), produced by intestinal L cells, its action on the pancreatic islets modified some aspects of insulin secretion pathophysiology. The scientific advances regarding GLP-1 and other gastro-intestinal hormones have certainly also turned a spotlight on the other cellular populations of the pancreatic islet. This reminded the world that the pancreas was not only made up of beta cells. This Special Issue was conceived to stimulate interest in a path parallel to that of beta cell/insulin, trying to understand if “the wingman” could ever reach the finish line first. The papers that contributed to the success of this Special Issue, in our opinion, have enriched our view on the pathophysiology of T2DM. Indeed, the contribution of each author has focused on new mechanisms of glucagon and pancreatic alpha cell actions in the context of the pancreatic islets and on extra-islet cellular targets.

Mohammad Sarif Mohiuddin et al., for example, described the importance of glucagon in the peripheral nervous system (PNS), focusing on its pleiotropic action in tissues that are usually not a common target of pancreatic hormones [7]. The original article by Tesi, M et al. highlighted and confirmed the observation that pancreatic islet endocrine cells are in dynamic balance [8]. A small number of pancreatic cells express more than one single hormone, and some cells may simultaneously contain both insulin and glucagon (Ins+/Glu+ cells). This latter β-/α-cell phenotype is prevalent, especially after islet exposure to cytokines, which induce resistance to apoptosis. These findings could open new perspectives not only for the prevention of pancreatic islet damage, but also in the field of regenerative medicine. As reported by Cefalo, C. and coworkers, the structural plasticity of the islets of Langerhans and the high level of intrinsic ribonuclease (RNase) activity in the organ are challenges for the isolation of different endocrine cell subtypes and harvesting high-quality RNA [9]. The authors confirmed laser capture microdissection (LCM) as the current preferable approach to isolate specific types of pancreatic cells and identified a specific RNA extraction protocol (Microkit/carrier) ensuring high-quality RNA that can be used for genetic and proteomic analysis. Filippello, A. and collaborators published original data using an in vitro model of alpha-cells chronically exposed to high levels of fatty acids [10]. This culture condition resulted in alpha-cells, insulin-resistance and glucagon secretion, both of which seem to be related to T2DM pathophysiology. Both diabetogenic effects were prevented by the use of D-chiro Inositolo (DCI), which shows an insulin-receptor sensitizer action on insulin signaling pathways and glucagon secretion. These results highlight the possibility that DCI could be a new option to improve the insulin sensitivity of pancreatic alpha-cells opening new perspectives for the treatment of T2DM patients showing hyperglucagonemia. A contribution for a better understanding of new aspects of the pathophysiology of T2DM comes from Gausseres, B. et al. [11]. Performing in vivo and ex vivo experiments, the authors found that the systemic lack of alfa 7 nicotinic receptor (α7-nAChR) induces metabolic alterations such as chronic mild high glycemia, impaired glucose tolerance, elevated plasma free fatty acid levels combined with deficit in β-cell mass and adipose tissue inflammation. These results show that α7-nAChR may play an important role in regulating glucose homeostasis and in modulating a novel pathway involved in T2DM onset. The Special Issue also includes two reviews summarizing well-known aspects of T2DM pathogenesis, which could open new horizons in diabetes research. Marrano, N. and his research group described similarities between GLP-1 and irisin, a myokine released in response to a high-fat diet and exercise focusing attention on the role of the gut, muscle and endocrine pancreas network on energy control [12]. Porcellati, F. et al. described the pathophysiological mechanisms of hypoglycemia and summarized the most recent advances in glucagon therapy, especially focusing on a new nasal formulation [13]. The authors hypothesize a new possible action of nasal glucagon administration in the brain and, as a consequence, in hepatic glucose release. This new potential pathway of glucagon action/modulation on the liver could result in a more advantageous severe hypoglycemia recovery and could represent an unexplored and novel aspect in the physiopathology of T2DM.

While in 1975 Unger hoped for a new role of glucagon not only as the “son of the servant” but as the main regulator of diabetes onset, and John Lennon imagined a world with more love and peace, today the authors of this Special Issue, almost 100 years after the discovery of glucagon, share a new vision: it is time for a new life of pancreatic islets, where each cellular entity can play a role in the integration and support of the secretory function of the endocrine pancreatic organ.

The final result of pancreatic cell–cell interaction could confer a leading role to each pancreatic cell component, including glucagon. According to this new point of view, hopefully in 2023, glucagon could be celebrated as a *“leading actor”* of pancreatic function instead of the *“son of the servant”.*

Right now, the time is not yet ripe, but we are cheering for *“the second”.*

Best wishes glucagon!

## Data Availability

Not applicable.
